# Investigating Depression and Anxiety among Turkish Immigrants with Endocrine Disorders Treated at the NPZR

**DOI:** 10.1192/j.eurpsy.2022.1429

**Published:** 2022-09-01

**Authors:** E.D. Cindik-Herbrüggen, R. Demirkol, Z. Demir, Ö. Ilıman

**Affiliations:** NPZR - Neuro-Psychiatrisches Zentrum Riem, Psychiatry/psychotheraphy, München, Germany

**Keywords:** Depression, Turkish immigrants, Endocrine diseases, Anxiety

## Abstract

**Introduction:**

Immigrants encounter difficulties in adapting to social life due to cultural and socioeconomic differences which consequently causes psychological and physical problems. Previous studies demonstrated that diabetes, high blood pressure, dyslipidemia and obesity are associated with psychological disorders.

**Objectives:**

This study aimed to investigate the frequency of depression and anxiety and to observe associated sociodemographic among endocrine patients treated at NPZR.

**Methods:**

190 Turkish psychiatric patients with at least one endocrine disorder (45.3% were male (n=86) and 54.7% were female (n=104) between the ages of 30-65, who participated in group therapy session at the NPZR, were recruited. Demographics, prevalence of depression and anxiety as well as current psychological conditions of participants were analyzed through Beck Depression Inventory, Hamilton Anxiety Scale, SCL-90-R and Personal Information Form.

**Results:**

The findings of our study demonstrated that prevalence of depression, anxiety and sleep disorders among Turkish immigrant patients with endocrine diseases is high. The mean scores of depression and anxiety were 31.39 and 32.61 respectively. The most common endocrine diseases were hypertension (51.6 %) and obesity (49.6%). Analysis of our research showed that there was no significant gender differences in the anxiety and depression scores. However, there was a significant relationship between income of participants and prevalence of anxiety, depression (p<0.05).

**Conclusions:**

The results of this research suggest that anxiety and depression disorders are highly prevalent among Turkish psychiatric patients with endocrine diseases Using the data of this study, the frequency of endocrine diseases among immigrant psychiatric patients can be analyzed.

**Disclosure:**

No significant relationships.

